# Maternal mental health and breastfeeding amidst the Covid-19 pandemic: cross-sectional study in Catalonia (Spain)

**DOI:** 10.1186/s12884-022-05036-9

**Published:** 2022-09-26

**Authors:** Marta Nicolás-López, Pablo González-Álvarez, Anna Sala de la Concepción, Maria Giralt-López, Beatriz Lorente, Inés Velasco, Paula Sol Ventura Wichner, Gemma Ginovart

**Affiliations:** 1 Department of Pediatrics, Neonatal Unit, Hospital Universitari Germans Trias I Pujol, Carretera de Canyet s/n, 08916 Badalona, Spain; 2 Department of Pediatrics, Neonatal Unit, Hospital Universitari Germans Trias I Pujol, Badalona, Spain; 3 Department of Paidopsychiatry, Hospital Universitari Germans Trias I Pujol, Badalona, Spain; 4 Department of Gynecology and Obstetrics, Hospital Universitari Germans Trias I Pujol, Badalona, Spain; 5 Research Institute Germans Trias I Pujol (IGPT), Badalona, Spain

**Keywords:** Breastfeeding, Maternal mental health, Bonding, Covid-19

## Abstract

**Background:**

Covid-19 pandemic became an unexpected stressor for the entire population and, particularly, for pregnant women and lactating mothers. The alarming infectious risk together with the lockdown period could affect the emotional state of mothers-to-be, as well as breastfeeding rates, mother-baby bonding, or neonatal weight gain. The aim of this study is to describe the impact of this world health emergency in mother-baby pairs right after the first wave of Sars-Cov-2 pandemic (from March to May 2020).

**Study design:**

A prospective observational study was carried out in mother–child dyads from those women who gave birth between June and August 2020 in a tertiary hospital. 91 mother-baby pairs were initially enrolled and 56 of them completed the follow-up. The study design had two separate steps: i) Step one: A clinical interview plus three psychometric tests (EPDS: Edinburgh Postnatal Depression Scale, PBQ: Postpartum Bonding Questionnaire and STAI-S: State-Trait Anxiety Inventory); ii) Step two: mother–child dyads were followed using a round of three brief telephone interviews (conducted at the newborn’s 7, 14 and 28 days of age) to accurately depict the newborn’s outcome in the neonatal period.

**Results:**

In terms of maternal mental health, 25% of the sample screens positively in the EPDS, requiring further evaluation to rule out depressive symptoms. STAI-state and PBQ detect no abnormalities in either anxiety levels or mother–child bonding in our sample, as 100% of the mothers score below the cut-off points in each test (34 and 26 respectively). When comparing feeding practices (breast/bottle feeding) in 2020 to those practices during pre-pandemic years (2017–2019), a significant increase in breastfeeding was found in pandemic times. All newborns in the sample showed an adequate weight gain during their first month of life.

**Conclusion:**

Women and newborns in our sample did not experience an increase in adverse outcomes in the neonatal period in terms of maternal mental health, breastfeeding rates, bonding and further neonatal development.

**Supplementary Information:**

The online version contains supplementary material available at 10.1186/s12884-022-05036-9.

## Introduction

The coronavirus disease 2019 (Covid-19) caused by the novel Sars-Cov-2 virus originated in Wuhan, China, towards the end of 2019, and was soon to be spread worldwide forcing the WHO to declare it as a pandemic on the 11th of March 2020. Several studies have demonstrated the impact of pandemics and natural disasters on the general population's physical and mental health [1–4], and the aftermath caused by COVID-19 is not an exception [5, 6].

The threat posed by the current pandemic has enforced many changes in medical care in a rapid and sudden way [7, 8]. Specifically in Neonatology and Obstetrics, the workforce was severely reduced due to relocation of staff to ICUs [9], and many pregnant women have had their routine appointments either cancelled or performed online [10]. Also, follow-up tests have been postponed, well-established support groups (i.e. breastfeeding groups, birthing and parenting courses) have been cancelled and restrictive clinical measures implemented to control Covid-19 transmission, including isolating the prospective mothers from their relatives during and after labour [11]. In addition, hospital stays were reduced, with mothers and their babies being discharged earlier to prevent nosocomial spread of the disease. This atypical scenario may have contributed to a different – and arguably more challenging – childbirth experience than in pre-pandemic times [12]. Such a stressful landscape exposed pregnant women to higher levels of emotional distress, potentially having a negative effect in the mother–child bonding process and the establishment of successful breastfeeding [13].

A wide range of maternal psychosocial factors (including racial disparity, socioeconomic disparities, lack of partner support and others) and their interrelationships has been identified as etiological factors likely to trigger antenatal stress and perinatal or postpartum depression among pregnant women [14]. However, the pandemic added a meaningful stressor not known so far.

On the other hand, when an infectious disease outbreak, epidemic, or pandemic occurs-particularly when it is associated with a novel pathogen-the question will naturally arise as to whether the pathogen can be transmitted through breastfeeding [15]. Beneficial effects of breastfeeding are widely known and have been highlighted in numerous studies [16, 17], having a positive impact in all developmental areas and serving as prevention for non-communicable diseases (NCDs) in adulthood such as obesity and metabolic syndrome, including diabetes, hypertension, and cardiovascular disease. Optimal nutrition during the first 1000 days is key to achieving the best development and health throughout life, and this constitutes a strategic period in terms of prevention and public health [18]. At the same time, failure to breastfeed can have a profound impact on maternal mood [19].

Traces of SARS-CoV-2 genetic material have been found in breastmilk, however, to date there is no evidence of transmission via breastfeeding [20, 21]. Therefore, experts encourage breastfeeding [22, 23] and have highlighted its important role for neonatal acquisition of protective antibodies against the Sars-Cov-2 virus [24, 25]. According to the World Health Organization (WHO) with the contribution of the European Pediatric Association-Union of National European Pediatric Societies and Associations and other main European Pediatric organizations, mothers with COVID-19 (or suspected COVID-19) can breastfeed their babies if they take appropriate precautions [26].

Here, we aim to evaluate the effects of the first wave of COVID-19 pandemic on the women who gave birth during the immediate post-lockdown period in a Maternity Hospital belonging to the Public Health System in Catalonia, in order to characterize the impact on mothers’ perinatal mental health, on the establishment of a successful mother–child relationship and on the newborn’s wellbeing during the first 28 days of their life [27].

## Study design and study population

A prospective observational study was carried out from June to August 2020 at Hospital Universitari Germans Trias i Pujol, located in Badalona (Barcelona). This is a Tertiary Hospital belonging to the Public Health System, which sees an average of nearly 2000 births per year.

Our hospital provides medical assistance to a multicultural and multiethnic population. More than a 30% of our patients are migrants from Asia and Africa, with the subsequent idiomatic barrier.

Participants were recruited during the first 12–24 h after labour, usually after the pediatrician’s first newborn assessment. Eligibility criteria included all healthy term (37—41.6 weeks) or late preterm (35.0 – 36.6 weeks of gestational age) newborns admitted to the Maternity Unit, regardless of the hours/days of hospitalization. All patients admitted to the Neonatal Unit immediately or shortly after birth, those with an important language barrier or those declining to participate were excluded.

Participants enrolled in a series of interviews conducted in two separate steps:

- In phase one, a survey (in Spanish) was handed to every mother agreeing to participate. Through a total of 36 questions we explored the following domains: i) the newborn’s future household depiction, in economic (current job situation and whether COVID-19 affected this, recent house moves) and emotional terms (expected or unexpected pregnancy and feelings around it), ii) breastfeeding intention, iii) psychiatric history: personal and family history of psychiatric disorders, previous/current psychiatric treatment, sleep disorders and substance abuse, iv)impact of COVID-19 in the patients’ lives (compatible symptoms experienced, previous diagnosis or contact with a confirmed positive, need of quarantine in the previous months) (the whole document can be found attached in Annex 1*).
- Additionally, three validated questionnaires were included: EPDS (Edinburgh Postnatal Depression Scale) [26], a subset of the 20 anxiety state questions of the STAI (State-Trait Anxiety Inventory) and the PBQ (Postpartum Bonding Questionnaire). Both EPDS and STAI have been validated and are widely used to explore perinatal anxiety [28], with shortened versions of the STAI-test having already been tested in pregnant women without major internal consistency changes [29]. Tests in the first round were either self-administered or answered by phone. Further information regarding each dyad’s medical history (course of pregnancy, type of labour and/or complications) was collected at this stage by the investigators.
- Phase two consisted of a round of three brief telephone interviews conducted when the newborn was 7, 14 and 28 days of age. Breastfeeding status, mother’s satisfaction with the aid received in establishing breastfeeding, the weight of the newborn and other questions relating to the health status of the child were asked, in an effort to accurately depict the outcome of the newborn in the neonatal period.
- As reference group to compare, data from our own hospital records were sourced for the same period of time in the three previous years (2017 to 2020), including exclusive breastfeeding percentages and other major neonatal outcomes in neonates admitted in the maternal area.

### Psychometric instruments

#### Edinburgh Postnatal Depression Scale (EPDS)

A questionnaire originally designed by Cox et al. [30] to screen for postpartum depression. It consists of 10 questions in which women are asked to rate how they have felt in the last 7 days prior to labour, with each question having 4 answers rating 0 to 3. A higher score is associated with a higher likelihood of depression. A cut-off of 10 or more points has been proposed as a marker of a high risk of postpartum depression for Spanish populations by previous studies [31]. Special importance was given to question 10, asking about the existence of suicidal thoughts, as stated before. The Spanish version of EPDS was used in our study.

### State-trait anxiety inventory (STAI)

A compound of 40 questions used to measure both *trait* anxiety, a predisposition to anxiety in different scenarios, and *state* anxiety, the degree of anxiety felt at a particular moment. It can be split consequently in two subsets of questions, STAI-T and STAI-S. Questions are scored using a 4-point Likert-type scale. As pregnancy entails a series of major changes in a woman’s life, STAI scores have been found to be higher in pregnant women when compared to the general population. Variations in the STAI-S have been documented through all trimesters of pregnancy, and have already been proposed as a useful tool to monitor the response to interventions aimed at lowering prenatal anxiety [32]. Higher scores correlate with greater anxiety. A study conducted amongst Portuguese women [33] found a cut-off of 34 to be the optimal value for anxiety screening during postpartum, a value we adopt in our study to approach the anxiety level in our population.

#### Postpartum bonding questionnaire (PBQ)

Described as the unique affective relationship established between mother and child in which warm, positive feelings of affection and care flourish, bonding is probably the most important process taking place in the postpartum [34]. Originally developed by Brockinton et al. [35], the PBQ aims at diagnosing bonding disorders through a set of 25 questions using a 6-point Likert-type scale. The sum scores range from 0 to 125 points across four scales (impaired bonding, rejection and anger, anxiety about care and incipient abuse) with a proposed cut-off value of 26, over which any of the previous bonding disorders may be present, with scores over 40 indicating severe bonding disturbances. These have been validated in the Spanish population showing no significant variations, thus being chosen as cut-offs in our analysis [36].

## Recruitment and statistical analysis

Recruitment started on the 1st of June 2020, almost three months after the COVID-19 pandemic outbreak in Spain, and closed on the 3rd of August 2020, just as the country transitioned from total lockdown (enforced from the 14th of March 2020 until the 1st of May 2020) into a series of progressive stepping-down stages intended to guide the population back to normality.

To ensure accurate assessments in psychometric scales and questionnaires, we establish a competent understanding of Spanish language as requirement.

All participants received comprehensive information regarding the aims and development of the study, and informed consent forms were signed as a pre-entry requirement. Sensitive information was obtained from the psychological tests used, with the EPDS of every participant being revised on collection to enable prompt psychiatric referral. Special attention was given to question no.10, in which it was asked about attempted self-harm and if so, early specialized assessment was started urgently.

Concerning the statistical analysis, sample size was calculated according to an observation compared to a reference ( in this case, we consider as standard a prevalence of 25% of score > 10 in Edimburgh scale): accepting a risc alfa 0.05 and beta below 0.2, in a bilateral contrast we need 21 subjects to detect a difference equal or superior to 0.5 units. A standard deviation of 0.5 was assumed and a rate of loss to follow-up of 10%.

Data were expressed as median [interquartile range] for continuous variables or as numbers/percentages for categorical variables. The contrast hypothesis for two samples was evaluated with the Student´s t-test for quantitative variables and Chi-squared test in cases of categorical ones. If variables did not adjust to normality, a Kruskall-Wallis test was done. Associations between breastfeeding, demographic variables and results of psychological test were estimated by univariable and multivariable logistic regression, and presented as odds ratios and 95% CI. Associations between STAI scores, EPDS, PBQ scores were estimated by univariable and multivariable linear regression, and presented as unstandardized regression coefficients and 95% CI. In all cases the rejection level for a null hypothesis was α < 0,05.

The Statistical Package for Social Sciences (SPSS) version 23.0 was used to perform the analyses.

## Results

**Fig. 1:**
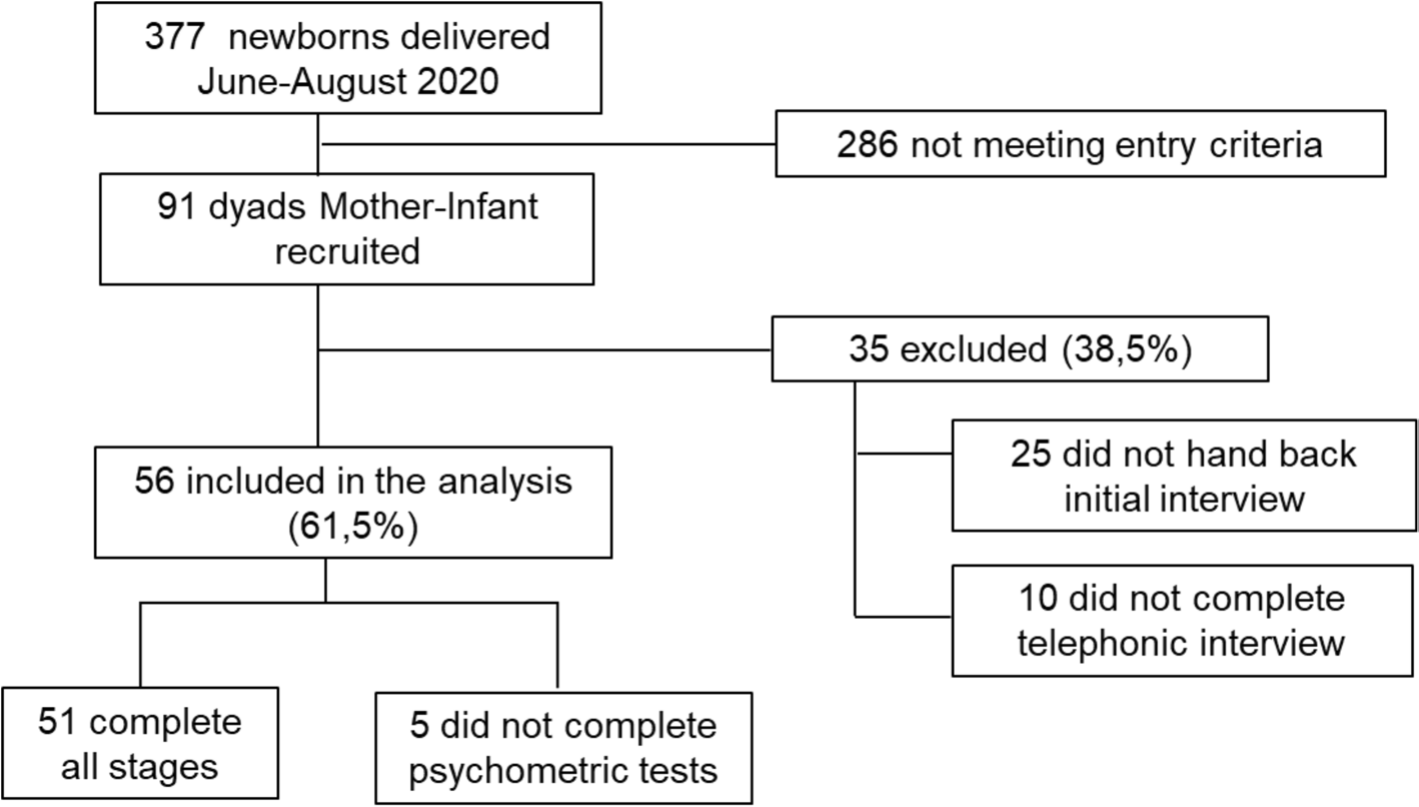
Flowchart of the sample’s selection

Over the observational period (1^st^ June 2020 – 3^rd^ August 2020), a total of 377 newborns were delivered in our hospital. Of these, 286 mother-infant dyads were excluded for language barriers or refusal to participate. 91 mother-infant dyads were recruited, with 56 (61%) included in the final analysis. A subset of 5 mothers in this group failed to complete either all or some psychological tests. However, given that they answered all phone interviews during follow-up we decided to include them where appropriate in the analysis. Amongst the remaining 35 (39%), 25 were ruled out during phase 1 due to their forms either coming back blank or being lost, with the other 10 failing to answer the phone at any of the interviews scheduled within the follow-up (Fig. 1).

Table 1 outlines the descriptive data of the sample. It summarizes the information obtained from the survey’s different domains, of which the following can be underlined: To start with, the pandemic at the time has seemingly had little or no effect on the majority of the participants’ economic situation (85%). Notably, the great majority of the pregnancies had been planned (82,1%), with only 2 (3,6%) mothers having considered an abortion. Almost all participants were in a stable relationship (55/56, 98,2%) and described their environments as supportive (54/56, 96,4%). As for breastfeeding, up to 49 (87,5%) mothers intended to exclusively breastfeed their children immediately after labour, of which 36 (64,3%) admitted to being satisfied with the information and assistance received. No significant burden of psychiatric disorders was found, with 14 mothers (25%) having first degree relatives diagnosed with psychiatric disorders and a total of 11 women (19,6%) having been prescribed psychiatric medication in the past. For the effects of the COVID-19 pandemic, none of the participants had been diagnosed with the disease (with all of them having been tested at least at the time of hospital admission), and 5 of them (8,9%) had lost close relatives due to this infection.

**Table 1:** Data collected from the survey organized by domains and descriptive analysis of the sample (*n* = 56)

**1. Household portrayal (economic and affective domains)**	**N (%)**^**a**^
**Mother's age, years.** Median [IQR]	34.3 [30–37.3]
**Mother’s job status/category**	
Housekeeper	5 (8.9)
Self-employed	1 (1.8)
Jobless	12 (21.4)
Disabled	1 (1.8)
Employed by others	37 (66.1)
**Effect of the pandemic on the mother's work status**	
No effect/has improved	48 (85.7)
Has worsened	8 (14.3)
**Current pregnancy planned**	46 (82.1)
**Abortion considered during present pregnancy**	2 (3.6)
**Pregnancy achieved through assisted reproduction techniques**	10 (17.9)
**History of previous maternal abortions**	13 (23.2)
**Voluntary abortions in previous pregnancies**	6 (10.7)
**Mother’s relationship status at the time of labour**	
Partnered	53 (96.4)
Single	2 (3.6)
**Quality of the relationship maintained with partner**	
Single	2 (3.6)
Overall good	51 (91.0)
Tense relationship, considering breakup	3 (5.4)
**Mother describes environment as supportive at the time of labour**	54 (96.4)
**Number of gestations.** Median	2.3
**Number of children (including the newborn)**	
One	28 (50.0)
Two	23 (41.0)
Three or more	5 (9.0)
**2. History of psychiatric disorders**	
**Average of sleep hours during current pregnancy**	
4-6 h	44 (78.5)
6-8 h	9 (16.1)
More than 8 h	3 (5.4)
**Maternal history of psychiatric drugs consumption**	11 (19.6)
**Maternal history of previous psychiatric hospitalization**	1 (1.8)
**Family history of psychiatric disorders (1st line relatives only)**	14 (25)
**Family history of suicide**	2 (3.6)
**Substance consumption during current pregnancy**	
Tobacco	7 (12.6)
Alcohol	2 (3.6)
Substance abuse	0 (0)
**3. Covid-19 status**	
**Maternal history of symptoms compatible with COVID-19 during current pregnancy**	24 (42.9)
**Cohabitants’ history of symptoms compatible with COVID-19**	8 (14.3)
**Pregnant women diagnosed with COVID-19 during current pregnancy/at labour**	0 (0)
**Cohabitants diagnosed with COVID-19 or needing quarantine**	2 (3.6)
**Next-of-kin or cohabitant deceased due to COVID-19**	5 (8.9)
**4. Perinatal data**	
**Gestational age, weeks.** Median [IQR]	39w1d [38w2d—40w2d]
**Apgar test score.** Median	9/10
**Anthropometric data of the newborn**	
Weight at birth, grams. Median [IQR]	3330 [2937–3675]
Length at birth, cm. Median [IQR]	49.5 [47–50]
Head circumference at birth, cm. Median [IQR]	34.5 [33.5–35.5]
**Satisfaction with the support received regarding EBF immediately after labour**	41 (73.2)
**Hospital stay**	
Early discharge (< 48 h)	12 (21.4)
Conventional (48-72 h)	36 (64.2)
Prolonged stay (> 72 h)	8 (14.4)
**Newborns admitted to Neonatology**	4 (7.1)
**Maternal postpartum complications**	
Fever	1 (1.8)
Hypertension	1 (1.8)
Anaemia	7 (12.5)
No complications	47 (83.9)

^a^ Results expressed in N (%) unless otherwise specified. *N* Absolute frequency, *IQR* Interquartile range

Table 2 summarizes the scores of the psychometric tests passed in Phase 1.

**Table 2:** Results of the psychometric tests in the sample. *N* = 51

**EPDS**		**N (%)**^a^
Total score	Median score [IQR]	5 [3–9]
	- < 10	40 (78.4)
	- ≥ 10	11 (21.6)
Question 10	- Negative (0 points)	50 (90)
	- Positive (1, 2 or 3 points)	1 (2)
**STAI**		
Total score	Median score [IQR]	8 [5–13]
	≤ 34	51 (100)
**PBQ**		
Total score	Median score [IQR]	4 [1–8]
	≤ 26	51 (100)

*N* Absolute frequency, *IQR* Interquartile range, *EPDS* Edinburgh Postnatal Depression Scale, *STAI* State-trait anxiety inventory, *PBQ* Postpartum bonding questionnaire

^a^ Results expressed in N (%) unless otherwise specified

EPDS results showed a mean score of 6, with 25% of the sample screening positively as they scored over the cut-off value of 10, requiring further evaluation to rule out depressive symptoms. Only 1 (1.8%) mother gave a positive answer to question 10 assessing for suicidal ideation, being promptly referred to the Psychiatric Department of our hospital for further evaluation.

STAI-*state* and PBQ detected no abnormalities in either anxiety levels or mother–child bonding in our sample, as 100% of the mothers scored below the cut-off points in each test (34 and 26 respectively).

There were solid correlations among scores obtained in the three tests: EPDS and STAI-*state* (*r* = 0,489; *p* < 0.001) and STAI-*state* and PBQ scores (*r* = 0,378; *p* = 0.006). Linear regression for STAI-*state* was predicted by EPDS (*p* = 0.002) and PBQ score (*p* = 0.036) (R^2^ = 0,306; *p* < 0.001). Mutiple regression and 95% CI of the unstandardized regression coefficients for psychometric tests were calculated (Suppl Table 1).

**Fig. 2:**
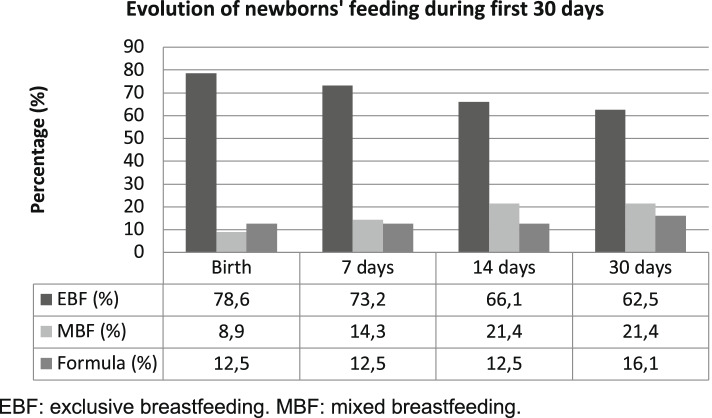
Type of feeding and weight increase in the first 30 days of life of the newborns in the sample. N = 56

Interviews in the second stage were conducted at 7, 14 and 28 days, recording the progression of exclusive breastfeeding (EBF) rates as shown in Fig. 2. A total of 9 mothers switched from EBF to other types of feeding: 7 added formula to their newborn’s diet while 2 stopped breastfeeding entirely. Therefore, in terms of breastfeeding out of the 87.5% of the mothers intending to breastfeed their infants at birth (78.6% exclusively and 8.9% mixed with bottle-feeding), 83.9% of women in the sample still practice some form of breastfeeding by the end of the observational period (Fig. 2).

Analysis of the motivation behind the type of feeding chosen showed the following results:

- At birth all 5/5 cases of mixed feeding started formula on medical recommendation, while 7/7 mothers opting not to breastfeed did so out of their own accord.
- At 7 days follow-up, 5 mothers in the EBF group added formula to their newborn’s diet (4/5 on medical advice), while 2 mothers in the mixed-feeding group went back onto EBF.
- Day 14 results in 5 more women switching from EBF to mixed-feeding, with only 1/5 doing so as instructed by her pediatrician. Also at this time, 1 mother in the mixed-feeding group stopped formula in favor of EBF.
- Finally, when interviewed on day 28, 3 mothers report having changed from EBF to mixed feeding (MBF) (2/3 doing so on medical advice), with 3 more in the MBF group stating changes: 1 going back to EBF and 2 leaving breastfeeding for good.

**Fig. 3:**
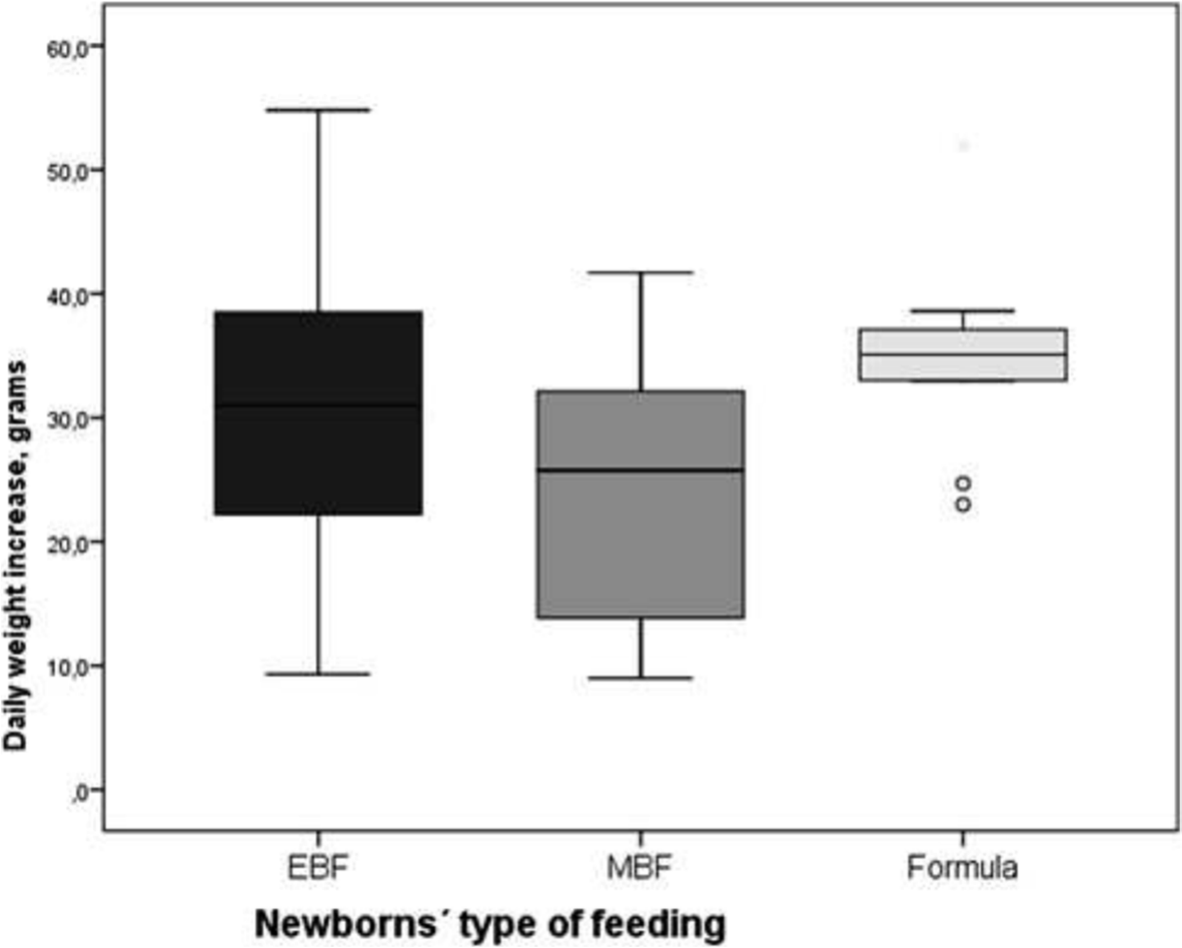
Daily weight increase analysis by subgroups in the first 30 days of life

Weight at 30 days of life was extracted from each newborn’s clinical history, therefore monitoring weight increase during their first month of life ([weight at 30 days of life – weight recorded at birth]/30 days). Results show an average of 31.4 g increase daily [21.9 – 37.5]. Subgroup analysis comparing weight increase between the different types of feeding recorded showed no significant differences (*p* = 0.09) as shown in Fig. 3.

Assessing the quality of the assistance received in the establishment of breastfeeding revealed that 19/56 women in the study (33.9%) considered themselves poorly informed when asked at birth. Particularly, out of the 7 women choosing formula feeding from birth, 3 considered the aid received insufficient. When asked again at the end of the study on day 30, 16/56 (28.5%) women were still not satisfied with the support received.

**Fig. 4:**
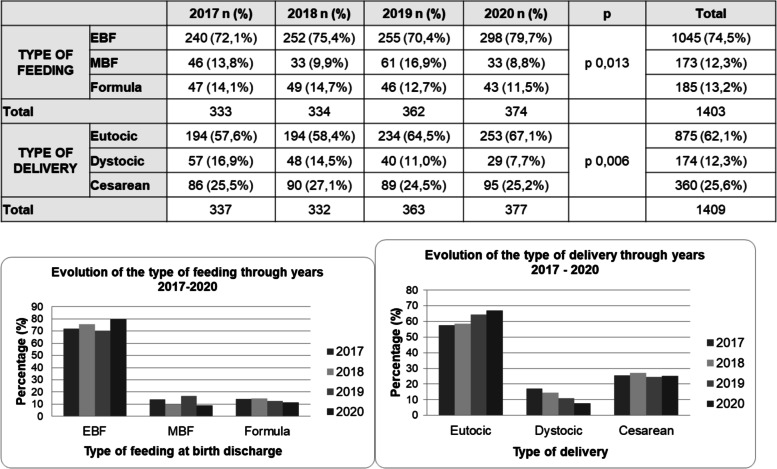
Type of feeding and type of delivery from June to August (years 2017–2020). EBF: exclusive breastfeeding. MBF: mixed breastfeeding

Previous breastfeeding rates from the past three years concerning the same period of time (June, July and August from years 2017 to 2020) were sourced from our hospital registry and are presented in Fig. 4. It is important to note that physicians fill in the registry only once, prior to the newborn’s discharge; thus, data in the registry reflects breastfeeding intention alone, but not its maintenance and variations. Also, information in the registry may not include all the variables of interest for all the newborns discharged, as shown by the disparity between the total amount of type of feedings (374) and deliveries registered (377). Missing entries in the type of feeding category represent 3/377 (< 1%), not compromising the validity of the registry.

From June to August 2020 a total of 377 babies were delivered in our centre, with women intending to practice some form of breastfeeding (EBF + mixed) adding up to a total of 331 (88.5%). A small variation was found from the proportion observed in our study (EBF + mixed = 87.5%), supporting the representativity of the sample. When comparing the rates of types of feeding between 2020 and each one of the previous years, significant variations are found in 2020 versus years 2017 and 2019 (*p* = 0.045 and p 0.003 respectively). A statistically significant rise is also found in breastfeeding rates in 2020 when compared to the aggregated proportions observed in the pre-pandemic years (2017 to 2019).

Univariable and multivariable logistic regression were calculated to identify potential demographic or clinical variables that might influence the choice of breast/bottle feeding. No association with the way of delivery, maternal occupation or existence of maternal pathology were found (data not shown).

In order to explore if the manner of delivery had been affected, Fig. 4 also includes the rates of delivery type observed (eutocic, dystocic and C-section). The figures show a significant reduction in the rates of dystocic delivery in the summer months of 2020 when compared to previous years. Equally, a statistically significant reduction in bottle-feeding rate is also found when comparing pre and post-pandemic periods.

## Discussion

Our study found that 25% of pregnant women were positively screened by the EPDS, even though they showed low levels of anxiety, with the entire sample scoring below the threshold in the STAI. Additionally, PBQ does not detect anomalies in the quality of bonding established amongst mother and child.

Feeding rates registered during the first months of the Covid-19 pandemic show not only steadiness but a significant increase in the number of mothers practicing any form of breastfeeding (EBF or MBF) when compared to pre-pandemic years. For babies, no major health issues were reported during follow-up and the average weight gain in their first month of life was adequate.

In our sample, the incidence of depressive symptoms in the immediate postpartum was slightly higher than previously observed in the Spanish population, with 25% screening positively in the EPDS (against 21% at 6 weeks postpartum reported in previous studies) [37, 38]. Pregnancy is a time of increased vulnerability to the development of depression.

The mental health of pregnant women affects the health outcomes of both the pregnant women and their infants [39]. Infants born to mothers who have experienced severe perinatal stress are reported to have altered behavioral responses to stress. Changes in the antenatal stress-related biology of pregnant women may be linked to adverse behavioral responses toward their infants [40]. In addition, depression during pregnancy is often associated with adverse birth outcomes, including preterm birth, low infant birth weight, and fetal growth restriction [41].

Other studies have reported high levels of anxiety and depression in pregnant and postpartum women during the COVID pandemic [42–44]. Some of the risk factors for anxiety or psychological distress have been identified: women with mental treatment history [42], those who received inadequate support for childbirth or felt unsafe at hospital [43], and those concerned about not getting the necessary prenatal care, relationship strain and social isolation [44]. On the contrary, higher levels of education [45] as well as perceived social support and support effectiveness were associated with fewer psychological symptoms [44]. In our sample, most participants had a partner and described their environments as supportive.

Rates and severity of postpartum depression (PPD) and anxiety symptoms among women seeking treatment for PPD have worsened during the COVID-19 pandemic [46]. Postpartum anxiety (PPA) and breastfeeding self-efficacy and bonding at the early postpartum period can be used to predict PPD [47]. Most PPD was the continuation of prenatal psychological problems and emotional disorders, indicating a significant correlation between prenatal psychological status and the occurrence of PPD [48]. Since the incidence of postpartum depression increases gradually after delivery, the assessment at birth may underestimate the real magnitude of the problem [49]. Although the second phase of our study (periodical phone calls) could have been useful to detect bonding disorders or late depressive symptoms, our study did not find significant altered scores in depressive or anxiety items.

A very recent multinational study found high levels of depressive symptoms and generalized anxiety among pregnant and breastfeeding women during the COVID-19 outbreak. The study findings underline the importance of monitoring perinatal mental health during pandemics and other societal crises to safeguard maternal and infant mental health [50].

Prior to the pandemic 90% of Catalan children were breastfed according to available epidemiological data [38], but the impact of COVID-19 on this had not been fully established so far. The rates of EBF at birth in our geographical area increased in the past 4 years. In interpreting this data, we must acknowledge the adherence of our hospital to the BFHI (Baby-friendly Hospital Initiative) ever since year 2018, an initiative that has the promotion of EBF amongst its main objectives. Ever since joining the scheme, our data show annual EBF numbers of > 75% on average. Nevertheless, the rise observed in the rates of breastfeeding in 2020 (both EBF alone and EBF and MBF altogether) is significantly higher despite the added difficulties of care the pandemic has brought along, and even, an increase in the percentage of mothers choosing to exclusively breastfeed their children (72,6% in pre-pandemic years versus 79,3% in the pandemic period). Average weight increase during the first 30 days of life shows an adequate linear growth, with over 75% of the sample gaining over 21.4 g daily. However, caution should be taken in interpreting these data, since all the newborns in the sample were born at term with a normal mean weight at birth.

Elevated posttraumatic stress, anxiety/depression, and loneliness are highly prevalent in pregnant and postpartum women across 64 countries during the COVID-19 pandemic. Excessive information seeking and worries related to children and medical care are associated with elevated symptoms, whereas engaging in hygiene-related preventive measures were not. In addition to screening and monitoring mental health symptoms, addressing excessive information seeking and women's worries about access to medical care and their children's well-being, and developing strategies to target loneliness (e.g., online support groups) should be part of intervention efforts for perinatal women [51].

In any case, our findings do not support our initial hypothesis postulating that the stress caused by the pandemic in pregnant women was going to have a negative effect on their newborn’s health, worsening women’s mood, altering the establishment of breastfeeding, and impairing the development of a solid mother–child bond. These unforeseen results may suggest that pregnant women took benefit from the standstill caused by the pandemic; perhaps the anticipated work-leave, the obligatory at-home rest and the increase in the time spent with their families and newborn babies, allowed them to bond and get to know each other [52].

Additionally, several care-related factors may have contributed to this positive effect. Maintaining women’s wellbeing was a priority from the beginning, and efforts were made to ease the experience of women in labor, allowing them to be accompanied by a person of their choice (usually their partners) throughout their stay [53]. Second, at-home visits 24 h after early discharge, carried out by expert midwives, helped to closely monitor the newborn, and enhanced the breastfeeding process despite the reduction in hospital stays [54]. Also, telephone interviews during the first 28 days of life carried out by a healthcare professional to monitor their child’s development may have provided a source of comfort and security for some parents.

We acknowledge some limitations to the study. First, a significant proportion of our population could not be included in the study. It seems clear that a bias of selection was introduced since the competence in Spanish was lacking in more than a 50% of potential participants. Sadly, we could not count on a cultural agent to translate the scales.

Additionaly, some patients refused to participate for logistical reasons (length of the questionnaire, frequency of calls in the follow-up). Second, comparisons are made using a larger historical cohort extracted from the hospital registry, including all births occurred in months of interest and presenting only static data (variables collected only once, at the time of birth, with no prospective analysis). Therefore, direct comparisons cannot be made, with differences serving only as starting points to formulate new hypotheses.

But our study also pointed out some negative aspects of our clinical practice. First, from our sample a total of 19 out of 56 (33.9%) of mothers claim to be misinformed with regards to breastfeeding when asked at birth, a proportion that remains almost unchanged 28 days after (16/56, 28.5%) [55]. Considering the many contacts with the healthcare system that these new mums had had, with an average of 3 medical visits (clinical and physical interview, anthropometric evaluation and dietary counselling) and at least one midwife visit, many opportunities to give accurate information on breastfeeding seem to be lost, highlighting a potential area of improvement. Given the importance of breastfeeding and its desired immunologic properties, all health care providers should use these opportunities to leverage breastfeeding as a critical intervention to improve health and developmental outcomes and save the lives of children around the world [56].

Second, postpartum represents a delicate time of transition, where psychological difficulties and disturbances are often encountered [54]; so, we advocate paying more attention to women’s health status in the immediate weeks after labour, especially in pandemic times. Whereas anxiety levels don’t seem to rise in response to the Covid-19 situation, screening for postpartum depression using the EPDS tested positive for a higher percentage of women than that described prior to the pandemic (25% vs 21%). Such findings suggest that the pandemic has an exacerbating effect on depressive symptoms, and, consequently, closer monitoring of mother’s mental status might benefit women and their newborns during postpartum [57–59].

Finally, attention should be given to practicalities to better deal with the difficulties imposed by the pandemic, while restrictions last: it would be advisable to reduce surveillance appointments to the minimum needed to ensure adequate healthcare. In order to safely do this, mother–child dyads should be provided with means of accessing information, asking doubts if needed or communicating the need for an appointment (other than being forced to consult to the emergency services). For this system to function properly, communication between primary care and specialized centers and amongst all the caregivers involved in the process (neonatologists, pediatricians, psychologists, obstetricians, midwives, nurses) would be of the utmost importance, working as a single force to efficiently reduce out-of-home visits.

To our knowledge this is the first study in our area of influence which has evaluated the impact of SARs-Cov-2 pandemic on maternal mental health (using three different psychometric scales) and their consequences in breastfeeding rates. Public health campaigns and medical care systems need to explicitly address the impact of COVID-19 related stressors on mental health in perinatal women, as prevention of viral exposure itself does not mitigate the pandemic's mental health impact [51].

## Conclusion

Women and newborns in our sample showed no signs of adverse outcomes in the neonatal period in terms of breastfeeding, bonding and weight gain, despite the adversities forced upon healthcare by the pandemic. Although maternal anxiety levels were seemingly unaffected, depression scores were higher than in pre-pandemic times. Conversely, breastfeeding rates were higher than those observed prior to the arrival of Sars-Cov-2 virus infection.

## Supplementary Information

**Additional file 1.** Annex 1.

**Additional file 2: Supplementary Table 1**. Factors associated with mental health test.

## Data Availability

All data generated or analysed during the corrent study are included in this published article.
