# Supplementary material for: Maternal mental health and breastfeeding amidst the Covid-19 pandemic: cross-sectional study in Catalonia (Spain)
Source: BMC Pregnancy Childbirth. 2022 Sep 26;22:733. doi: 10.1186/s12884-022-05036-9 (PMC9511438; doi:10.1186/s12884-022-05036-9)
Supplement: Supplementary file 1 — Additional file 1. Annex 1. [file 12884_2022_5036_MOESM1_ESM.zip › ANEX 1.1.pdf]

¿Cómo se siente respecto a su bebé?		Siempre	Muy a menudo	Bastante a menudo	A veces	Raramente	Nunca
1.	Me siento cercana.						
2.	Desearía que volvieran los días en que no lo tenía.						
3.	Me siento distante.						
4.	Me encanta abrazarlo.						
5.	Lamento haberlo tenido.						
6.	Siento como si no fuera mío.						
7.	Siento que me toma el pelo.						
8.	Lo quiero con locura.						
9.	Me siento feliz cuando ríe.						
10.	Me irrita.						
11.	Disfruto jugando con él.						
12.	Llora demasiado.						
13.	Me siento atrapada como madre.						
14.	Me siento enfadada con mi bebé.						
15.	Siento resentimiento hacia él.						
16.	Es el más guapo del mundo.						
17.	Desearía que de alguna manera desapareciera.						
18.	He hecho cosas que son perjudiciales para él.						
19.	Me pone nerviosa.						
20.	Me asusta.						
21.	Me fastidia.						
22.	Me siento segura cuando lo estoy cuidando.						
23.	Siento que la única solución es que otra persona lo cuide.						
24.	Tengo ganas de hacerle daño.						
25.	Se consuela fácilmente.						

STAI - Estado

A continuación encontrará unas frases que se utilizan corrientemente para describirse uno a sí mismo. Lea cada frase y señale la puntuación 0 a 3 que indique mejor cómo se SIENTE Vd. AHORA MISMO, en este momento. No hay respuestas buenas ni malas. No emplee demasiado tiempo en cada frase y conteste señalando la respuesta que mejor describa su situación presente.

	Nada	Algo	Bastante	Mucho
1. Me siento calmada				
2. Me siento segura				
3. Estoy tensa				
4. Estoy contrariada				
5. Me siento cómoda (estoy a gusto)				
6. Me siento alterada				
7. Estoy preocupada ahora por posibles desgracias futuras				
8. Me siento descansada				
9. Me siento angustiada				
10. Me siento confortable				
11. Tengo confianza en mí misma				
12. Me siento nerviosa				
13. Estoy desasosegada				
14. Me siento muy "atada" (como oprimida)				
15. Estoy relajada				
16. Me siento satisfecha				
17. Estoy preocupada				
18. Me siento aturdida y sobreexcitada				
19. Me siento alegre				
20. En este momento me siento bien				
