# Supplementary material for: Maternal mental health and breastfeeding amidst the Covid-19 pandemic: cross-sectional study in Catalonia (Spain)
Source: BMC Pregnancy Childbirth. 2022 Sep 26;22:733. doi: 10.1186/s12884-022-05036-9 (PMC9511438; doi:10.1186/s12884-022-05036-9)
Supplement: Supplementary file 1 — Additional file 1. Annex 1. [file 12884_2022_5036_MOESM1_ESM.zip › ANEX 1.2.pdf]

Datos perinatales:

ID: …………………………………………….

1. Patología previa de la madre:
   ( ) HTA
   ( ) Diabetes no gestacional
   ( ) Obesidad
   ( ) Enfermedad autoinmune
   ( ) Otras: ……………………….

2. Patologías durante el embarazo actual:
   ( ) Preeclampsia
   ( ) Diabetes gestacional
   ( ) CIR
   ( ) Corioamnionitis
   ( ) Otras: ……………………….

3. Número de gestaciones de la madre:

4. Número de abortos de la madre:

5. Edad gestacional (en días):

   5.1. Edad gestacional (en semanas):

6. Tipo de parto:
   ( ) Eutócico
   ( ) Vacuum
   ( ) Fórceps
   ( ) Cesárea

7. Inducción del parto (sí/no):

8. Tipo de anestesia durante el parto:
   ( ) Ninguna
   ( ) Peridural
   ( ) Intradural
   ( ) Local
   ( ) General

9. Apgar en 1º minuto de vida:

10. Apgar en 5º minuto de vida:

11. ¿Se precisó reanimación neonatal? (sí/no):

12. Sexo del recién nacido (femenino/masculino):

13. Peso del recién nacido (en gramos):

14. Longitud del recién nacido (en centímetros):

15. Perímetro craneal al nacimiento (en centímetros):

16. ¿Requirió ingreso en Unidad Neonatal (sí/no)?

17. Complicaciones postparto de la madre:
    ( ) Sangrado
    ( ) HTA
    ( ) Fiebre
    ( ) Otras…………………

18. Etnia a la que pertenece la madre:
    ( ) Europea
    ( ) Gitana
    ( ) Hindú
    ( ) Latinoamericana
    ( ) Asiática
    ( ) Africana
    ( ) Otras…………………

Resultado de las encuestas realizadas:

1. Cuestionario de Whooley en 3º trimestre de la gestación actual:
    ( ) Negativo (ninguna respuesta afirmativa)
    ( ) Positivo (alguna respuesta afirmativa)

    Si Whooley positivo, puntuación total de la EPDS en 3º trimestre:

    Resultado en la pregunta 10 del EPDS en 3º trimestre:
    ( ) Negativa (0 puntos)

(   ) Positiva (1, 2 o 3 puntos)

2. Puntuación total en EPDS en el postparto inmediato:

   Resultado en la pregunta 10 del EPDS en 3º trimestre:
   (   ) Negativa (0 puntos)
   (   ) Positiva (1, 2 o 3 puntos)

3. Puntuación total en el test STAI (ansiedad estado) en el postparto inmediato: …………………………….

4. Puntuación en el test PBQ sobre vínculo madre-bebé en el postparto inmediato:

   ……………………………………………
