# Supplementary material for: Maternal mental health and breastfeeding amidst the Covid-19 pandemic: cross-sectional study in Catalonia (Spain)
Source: BMC Pregnancy Childbirth. 2022 Sep 26;22:733. doi: 10.1186/s12884-022-05036-9 (PMC9511438; doi:10.1186/s12884-022-05036-9)
Supplement: Supplementary file 1 — Additional file 1. Annex 1. [file 12884_2022_5036_MOESM1_ESM.zip › ANEX 1.pdf]

Lactancia materna y vínculo durante la pandemia COVID-19

Cuestionarios

Germans Trias i Pujol
Hospital

ID: ....

Telf: ....

Hospital Germans Trias i Pujol

Badalona, 2020

Escala de Edimburgo para la Depresión postparto

Paciente número: ____

Fecha: __________________________________

Si se encuentra en la etapa de postparto: número de semanas de postparto: _________

Como va a tener un bebé (o acaba de tener un bebé) nos gustaría saber cómo se ha encontrado emocionalmente. Marque la respuesta que más se acerca a cómo **se ha sentido en los últimos 7 días,** no solamente cómo se siente hoy. En el ejemplo que se encuentra a continuación, la "X" significa "Me he sentido contenta casi siempre durante la última semana".

EJEMPLO:

Me he sentido contenta:

_____ Sí, siempre

__X_ Sí, casi siempre

_____ No muy a menudo

_____ No, nunca

Complete las siguientes preguntas de la misma manera.

En los últimos 7 días:

1. He podido reír y ver el lado bueno de las cosas:

_____ Tanto como siempre

_____ No tanto ahora

_____ Mucho menos

_____ No, no he podido

2. He mirado al futuro con placer:

_____ Tanto como siempre

_____ Algo menos de lo que solía hacer

_____ Definitivamente menos

_____ No, nada

3. Me he culpado sin necesidad cuando las cosas marchaban mal:

_____ Si, casi siempre

_____ Sí, algunas veces

_____ No muy a menudo

_____ No, nunca

4. He estado ansiosa y preocupada sin motivo:

_____ No, nada

_____ Casi nada

_____ Sí, a veces

_____ Sí, a menudo

5. He sentido miedo o pánico sin motivo alguno:

_____ Sí, bastante

_____ Sí, a veces

_____ No, no mucho

_____ No, nada

6. Las cosas me oprimen o agobian:

_____ Sí, casi siempre

_____ Sí, a veces

_____ No, casi nunca

_____ No, nada

7. Me he sentido tan infeliz, que he tenido dificultad para dormir:

_____ Sí, casi siempre

_____ Sí, a menudo

_____ No muy a menudo

_____ No, nada

8. Me he sentido triste o desgraciada:

_____ Sí, casi siempre _

____ Sí, bastante a menudo _

____ No muy a menudo

_____ No, nada

9. He estado tan infeliz que he estado llorando:

_____ Sí, casi siempre

_____ Sí, bastante a menudo

_____ Solo ocasionalmente

_____ No, nunca

10. He pensado en hacerme daño a mi misma:

_____ Sí, bastante a menudo

_____ Sí, a menudo

_____ Casi nunca

_____ No, nunca

Test del vínculo en el postparto (PBQ)

Por favor, indique con qué frecuencia le ocurre lo que se detalla a continuación. No hay respuestas “buenas” o “malas”. Elija la respuesta que le parezca más adecuada a su experiencia actual:

Datos sociodemográficos

Le agradeceríamos su participación rellenando los siguientes campos:
ID:...........................

Fecha de nacimiento MADRE:

Fecha de nacimiento HIJO:

1. ¿Actualmente tiene pareja?
   Sí [ ] No [ ]

2. Número de hijos:

3. Marque su situación laboral:
   [ ] Ama de casa
   [ ] Estudiante
   [ ] Trabajadora por cuenta ajena
   [ ] Desempleo
   [ ] Invalidez
   [ ] Ns/Nc

4. En caso de haber seleccionado "desempleo", ¿Actualmente cobra prestación de desempleo?
   Sí [ ] No [ ]

5. Seleccione su categoría de trabajo:
   [ ] Directivo/a de la adminsitración y empresas. Técnico superior
   [ ] Profesional liberal, empresario o autónomo
   [ ] Administrativos/as, funcionarios/as en general. Mandos intermedios
   [ ] Dependientes/as de comercio y trabajadores/as de servicios, trabajador manual cualificado
   [ ] Trabajador/a manual no cualificado de industria, comercio o servicios
   [ ] Sin trabajo remunerado
   [ ] Ns/Nc

6. Seleccione la situación laboral de SU PAREJA:
   [ ] Ama de casa
   [ ] Estudiante
   [ ] Trabajadora por cuenta ajena
   [ ] Desempleo

[ ] Invalidez
[ ] Ns/Nc

7. En caso de haber seleccionado “desempleo”, ¿Actualmente SU PAREJA cobra prestación de desempleo?
   Sí [ ] No [ ]

8. Seleccione la categoría de trabajo de SU PAREJA:
   [ ] Directivo/a de la adminsitración y empresas. Técnico superior
   [ ] Profesional liberal, empresario o autónomo
   [ ] Administrativos/as, funcionarios/as en general. Mandos intermedios
   [ ] Dependientes/as de comercio y trabajadores/as de servicios, trabajador manual cualificado
   [ ] Trabajador/a manual no cualificado de industria, comercio o servicios
   [ ] Sin trabajo remunerado
   [ ] Ns/Nc

9. ¿Qué tipo de contrato tiene USTED actualmente?:
   [ ] Fijo/indefinido
   [ ] Temporal
   [ ] Prácticas
   [ ] Por obra o servicio
   [ ] Autónomo
   [ ] Sin contrato
   [ ] Ns/Nc

10. ¿Cómo ha influido la pandemia en su situación laboral?
    [ ] No me ha supuesto ningún cambio
    [ ] Ha cambiado para mejorar
    [ ] Ha cambiado para empeorar
    [ ] He tenido problemas laborales importantes
    [ ] No trabajaba fuera de casa

11. ¿Ha cambiado de domicilio recientemente o piensa cambiar?
    [ ] Sí
    [ ] No

12. ¿Se siente apoyada por su familia y entorno cercano?
    [ ] Sí
    [ ] No

13. ¿Ha interrumpido voluntariamente algún embarazo previo?
    [ ] Sí
    [ ] No

14. ¿Ha tenido algún embarazo deseado que no haya llegado a su fin?
    [ ] Sí
    [ ] No

15. ¿Cómo se sintió anímicamente en los postpartos anteriores? Considerando los primeros 6 meses después del parto:
    [ ] Éste es mi primer embarazo
    [ ] Estuve bien, me encontré bien de ánimo
    [ ] Me sentí triste y/o alterada pero no consulté por ello
    [ ] Me sentí deprimida y estuve en tratamiento
    [ ] Ns/Nc

16. ¿El embarazo actual fue planificado?
    [ ] Sí, fue planificado y bien recibido
    [ ] No fue planificado pero fue bien acogido
    [ ] No, fue un accidente
    [ ] Ns/Nc

17. ¿Ha buscado ayuda para quedarse embarazada?
    [ ] No
    [ ] Sí, he recibido ayuda mediante técnicas de reproducción asistida

18. ¿Se planteó en algún momento la posibilidad de interrumpir el embarazo?
    [ ] Sí
    [ ] No

19. ¿Qué relación ha mantenido con su pareja los últimos meses?
    [ ] No tengo pareja
    [ ] Una relación cálida y tierna
    [ ] Buena relación, con algunos desacuerdos puntuales
    [ ] Una relación fría, con una cierta tensión
    [ ] Una relación con tensiones constantes, planteamientos de ruptura o separación
    [ ] Ns/Nc

20. Actualmente, ¿está dando el pecho a su bebé?
    [ ] Sí
    [ ] No

21. Si NO le está dando el pecho, ¿por qué motivo?
    [ ] Por decisión propia
    [ ] Por indicación médica

22. ¿Cree que ha recibido información y apoyo suficiente en relación con la lactancia?
    [ ] Sí
    [ ] No

23. ¿En algún momento de su vida ha recibido tratamiento médico por depresión, nervios, insomnio u otros problemas emocionales?
    [ ] Sí
    [ ] No

24. ¿Toma actualmente alguna medicación?
    [ ] Sí Nombre:.........................
    [ ] No

25. ¿En algún momento de su vida ha tenido que estar ingresada por problemas psiquiátricos?
    [ ] Sí
    [ ] No

26. En su familia nuclear (padres y hermanos), ¿alguien ha sido diagnosticado de alguna enfermedad psiquiátrica (esquizofrenia, trastorno bipolar, depresión, ansiedad)?
    [ ] Sí ¿Quién, y de qué enfermedad? …………………………..
    [ ] No

27. ¿Ha habido algún suicidio en la familia? (padres, hermanos, tíos o primos)
    [ ] Sí
    [ ] No

28. Indique si ha consumido alguna de las siguientes durante el embarazo y con qué frecuencia:

	**Esporádica**	**1 vez/semana**	**Más de 1 vez/semana**	**No consume**	**Ns/Nc**
**Tabaco**					
**Alcohol**					
**Cannabis**					
**Cocaína**					
**Éxtasis**					
**Otros**					

29. Indique si consume alguna de las siguientes sustancias ACTUALMENTE:

	Esporádica	1 vez/semana	Más de 1 vez/semana	No consume	Ns/Nc
Tabaco					
Alcohol					
Cannabis					
Cocaína					
Éxtasis					
Otros					

30. Indique la cantidad de horas de sueño que suele dormir de forma habitual:

	Antes del embarazo	Actualmente
Entre 4-6 horas		
Entre 6-8 horas		
8 horas o más		

31. ¿Ha tenido alguno de los siguientes síntomas durante el embarazo?
    [ ] Dolor de cuello
    [ ] Fiebre
    [ ] Tos
    [ ] Dolor de articulaciones/de huesos
    [ ] Ahogo/dificultad para respirar
    [ ] Diarrea
    [ ] Anosmia (pérdida del sentido del olfato)

32. ¿Alguno de sus convivientes ha sufrido la sintomatología anterior?
    [ ] Sí
    [ ] No

33. ¿Ha sido diagnosticada de COVID-19 durante el embarazo?
    [ ] Sí
    [ ] No

34. En caso afirmativo, ¿requirió ingreso hospitalario?
    [ ] Sí
    [ ] No

35. ¿Alguno de sus convivientes ha sido diagnosticado de COVID-19 o ha requerido medidas de aislamiento?
    [ ] Sí
    [ ] No

36. ¿Alguien de su entorno cercano ha fallecido por COVID-19?
    [ ] Sí
    [ ] No
