# Supplementary material for: Maternal mental health and breastfeeding amidst the Covid-19 pandemic: cross-sectional study in Catalonia (Spain)
Source: BMC Pregnancy Childbirth. 2022 Sep 26;22:733. doi: 10.1186/s12884-022-05036-9 (PMC9511438; doi:10.1186/s12884-022-05036-9)
Supplement: Supplementary file 2 — Additional file 2: Supplementary Table 1. Factors associated with mental health test. [file 12884_2022_5036_MOESM2_ESM.docx]

|  | EPDS Test | | | STAI Test | | | PBQ Test | | |
| --- | --- | --- | --- | --- | --- | --- | --- | --- | --- |
|  | B | (95% IC) | | B | 95% IC | | B | 95% IC | |
|  |  | Lower Bound | Upper Bound |  | Lower Bound | Upper Bound |  | Lower Bound | Upper Bound |
| Breastfeeding | 1,148 | -2,633 | 4,930 | 1,578 | -3,793 | 6,948 | 1,180 | -3,858 | 6,217 |
| Maternal age (y) | -,106 | -,345 | ,134 | -,016 | -,360 | ,328 | -,046 | -,368 | ,276 |
| Professional activity (yes/no) | 2,699 | -,539 | 5,936 | -1,520 | -6,263 | 3,223 | -,062 | -4,528 | 4,405 |
| Sons (n) | ,585 | -1,348 | 2,517 | -1,757 | -4,452 | ,938 | ,064 | -2,517 | 2,646 |
| Misbirth (n) | ,635 | -1,338 | 2,608 | ,734 | -2,072 | 3,541 | ,532 | -2,100 | 3,164 |
| Caesarea | -2,003 | -5,607 | 1,602 | -1,882 | -7,049 | 3,285 | -2,513 | -7,314 | 2,288 |
| Gestational age  (weeks) | -,124 | -1,208 | ,960 | -,662 | -2,187 | ,862 | ,024 | -1,419 | 1,466 |
| Maternal postpartum complications | 1,685 | -2,230 | 5,599 | 1,531 | -4,060 | 7,123 | -1,759 | -6,984 | 3,465 |
| STAI Test | ,247 | **,027** | **,467** | N/A | N/A | N/A | ,265 | -,035 | ,564 |
| EPDS Test | N/A | N/A | N/A | ,497 | **,054** | **,940** | ,060 | -,382 | ,503 |
| PBQ Test | ,034 | -,216 | ,284 | ,302 | -,039 | ,643 | N/A | N/A | N/A |
| European | -2,880 | -6,388 | ,628 | -1,775 | -6,905 | 3,355 | -,609 | -5,440 | 4,222 |
| Chronic maternal somatic illness | ,776 | -2,225 | 3,778 | -1,255 | -5,512 | 3,001 | 3,182 | -,679 | 7,044 |

Supplementary Table 1: Factors associated with mental health test

Note: the bold numbers indicate the 95% CI of the unstandardized regression coefficients of the adjusted analyses that do not include the null. Abbreviations: B, unstandardized regression coefficients; CI confidence interval; N/A, not available. Adjusted by table variables
